# P02.195. Acupuncture for acute pain management in an emergency department: an observational study

**DOI:** 10.1186/1472-6882-12-S1-P251

**Published:** 2012-06-12

**Authors:** T Zhang, D Smit, D Taylor, S Parker, C Xue

**Affiliations:** 1 RMIT University, Australia, Melbourne, Australia; 2 Alfred Hospital, Melbourne, Australia; 3 Austin Hospital, Melbourne, Australia

## Purpose

To evaluate the impact on clinical outcomes by providing acupuncture as an option for acute pain management in the emergency department (ED) of a major metropolitan hospital in Melbourne, Australia.

## Methods

Adult patients (aged 18+ years) with acute pain attending the ED were initially assessed by medical staff and/or acupuncture staff for the suitability of acupuncture treatment. A total of 200 patients received acupuncture treatment. The selection of points for treatment was based on Chinese medicine diagnosis and theory. The treatment duration was 30 minutes on average with or without electrical stimulation. A Visual Analogue Scale (VAS) to measure pain scores before and after acupuncture treatment was the primary outcome.

## Results

Pain in the abdominal or flank region and muscle and musculoskeletal related pain were the common conditions treated (83%). A significant difference in the scores of before (mean = 7.01) and post treatment (mean = 4.72) was observed [t(193) = 14.81, p <.001]. Four patients (2%) reported adverse events during the course of the acupuncture treatment. These included two cases of slight bleeding with the other two cases of mild pain at the needling sites. No serious adverse events were reported. More than half (52.5%) of patients indicated that they would definitely consider acupuncture in the future for the same health condition and a further 31.8% said they probably would consider acupuncture again.

## Conclusion

Acupuncture can be an effective and safe adjunct intervention for patients with acute pain in settings such as the emergency pain management environment.

